# Sensitivity to Inflectional Morphemes in the Absence of Meaning: Evidence from a Novel Task

**DOI:** 10.1007/s10936-019-09629-y

**Published:** 2019-02-21

**Authors:** Luca Cilibrasi, Vesna Stojanovik, Patricia Riddell, Douglas Saddy

**Affiliations:** 1 Faculty of Arts, Charles University, Prague, Czech Republic; 2 Cambridge Language Sciences, University of Cambridge, Cambridge, UK; 3 School of Psychology and Clinical Language Sciences, University of Reading, Reading, UK

**Keywords:** Morphophonology, Inflectional morphology, Nonwords, Minimal pairs

## Abstract

A number of studies in different languages have shown that speakers may be sensitive to the presence of inflectional morphology in the absence of verb meaning (Caramazza et al. in Cognition 28(3):297–332, 1988; Clahsen in Behav Brain Sci 22(06):991–1013, 1999; Post et al. in Cognition 109(1):1–17, 2008). In this study, sensitivity to inflectional morphemes was tested in a purposely developed task with English-like nonwords. Native speakers of English were presented with pairs of nonwords and were asked to judge whether the two nonwords in each pair were the same or different. Each pair was composed either of the same nonword repeated twice, or of two slightly different nonwords. The nonwords were created taking advantage of a specific morphophonological property of English, which is that regular inflectional morphemes agree in voicing with the ending of the stem. Using stems ending in /l/, thus, we created: (1) nonwords ending in potential inflectional morphemes, vɔld, (2) nonwords without inflectional morphemes, vɔlt, and (3) a phonological control condition, vɔlb. Our new task endorses some strengths presented in previous work. As in Post et al. (2008) the task accounts for the importance of phonological cues to morphological processing. In addition, as in Caramazza et al. (1988) and contrary to Post et al. (2008), the task never presents bare-stems, making it unlikely that the participants would be aware of the manipulation performed. Our results are in line with Caramazza et al. (1988), Clahsen (1999) and Post et al. (2008), and offer further evidence that morphologically inflected nonwords take longer to be discriminated compared to uninflected nonwords.

## Introduction

### Regular, Irregular and Quasi-Regular Inflection

There has been a large body of research in psycholinguistics dealing with the processing of inflected verbs (Joanisse and Seidenberg 1999; Marslen-Wilson and Tyler 1997; Pinker 1991; Bybee and Slobin 1982; Kuczaj 1977; Stowell 2007; Jackendoff 1975; Seidenberg and Plaut 2014). According to one view, regular and irregular verbs differ in that irregular verbs are processed and stored as units, whereas regular verbs may be dealt with by (de)composing them into stems and affixes (Marslen-Wilson and Tyler 1997; Pinker and Ullman 2002; Post et al. 2008). Seminal work by Aronoff (1976), Halle (1973) and Jackendoff (1977) focused on production rather than perception and this research identified inflectional morphology as a form of syntax within words. Jackendoff (1975) proposes a distinction between regular and irregular inflection: while irregular forms are all stored as separate entries, regular forms are formed through rules that resemble the rules proposed for syntax. In particular, the combination of a stem and an affix resembles the basic operation of our syntactic system, described by Chomsky (1995) with the term *merge*. Psycholinguistic data have shown that this combinatorial rule-like explanation of regular inflectional morphology may be correct and that it may apply also to perception. In their set of experiments, Stanners et al. (1979) showed that stems of regular verbs are good primes for their inflected forms, while stems of irregular verbs are less effective primes of their inflected forms. This suggests that regular verbs are stored using stems and adding inflectional morphemes, while irregular forms are stored with several different entries, one for the stem and one for each inflected form. Discussing data from different studies, Pinker and Prince (1994) noted that, while reaction times for the recognition of irregular verbs is strongly related to the frequency of their form in the lexicon, reaction times for the recognition of regular verbs are less dependent on that measure. This suggests, according to the authors, that while for irregular forms the whole inflected form may be stored in the lexicon, for regular forms the stem may be stored alone.

Child-language studies have also showed the presence of rule-like phenomena, particularly in production. In an influential publication, Marcus et al. (1992) showed that children consistently create over-regularised forms of verbs. This type of pattern can easily be explained by assuming that children are applying a rule to any item that can be classified as a verb. Later in development, children store the irregular inflected forms and block the activation of regular inflections. In recent work on the developmental disorder known as Grammatical-Specific Language Impairment (Gra-SLI), van der Lely and Pinker (2014) have shown that the production of regular verbs can be impaired in a dissociated fashion from the production of irregular verbs, again suggesting the existence of a rule-based system for morphology, at least in production.

Neurological and neurocognitive evidence has corroborated the claim that regular and irregular forms require two distinct mechanisms, and that the mechanism for regular forms is a rule-like process. In a study on perception, Marslen-Wilson and Tyler (1997) report the case of two aphasic patients with acquired neurological damage showing impairment to the processing of regular inflected verbs, but who have intact performance on irregular inflected verbs. The participants were tested with a priming task, in which the inflected form was used to prime the non-inflected form. Age-matched participants showed priming for both types of verbs (regular and irregular forms) while the two patients showed priming only for the irregular forms. This finding suggests a distinction between regular and irregular inflection and it suggests that regulars and irregulars are supported by different neural systems (Marslen-Wilson and Tyler 1997, p. 592). This result justifies the idea of a qualitatively different system for the two types of verbs (namely, a rule-like system for regulars and a storing-based explanation for irregulars).

The development of electrophysiological and neuroimaging techniques has provided further evidence for these claims. In a functional Magnetic Resonance Imaging (fMRI) study performed on speakers of English, Tyler et al. (2005) showed that the perception of regularly inflected forms, in comparison to irregular forms, requires the activation of an extensive fronto-temporal network of the brain. The result was obtained with a minimal pairs judgment task performed on non-impaired adults. This result suggests that the human brain deals differently with regular and irregular forms, most likely by using rules for regular forms. A study conducted by Newman et al. (2007) with Event Related Potentials (ERPs) confirmed the finding of Tyler et al. (2005). In this experiment, participants were presented with regular and irregular verbs inflected in the past. Crucially, the past-tense was either well-formed or it contained a violation. When the violation was applied to an irregular verb, participants generated a brain component called P600, while when the violation was applied to a regular verb, participants additionally generated a brain component called Left Anterior Negativity (LAN). The LAN component is normally associated with grammatical compositionality and structure building (see Friederici 2002; Ullman 2001). This suggests that a violation of a regular verb corresponds to a violation of a morphosyntactic rule, while violation of an irregular verb does not entail any morphosyntactic rule violation. Pinker and Ullman (2002) propose that the differences observed between regular and irregular forms are due to the fact that the two types of verbs rely on qualitatively different systems. While (p. 464) “irregular forms are stored in the lexicon, a division of declarative memory, regular forms can be computed by a concatenation rule, which requires the procedural system”. According to this view, production takes place by means of a compositional rule, in which an affix is added to the stem, while perception takes place by a mirroring decompositional rule, in which an affix is stripped from the inflected form.

This type of account is often seen as contrasting to the so-called unit-based accounts (McClelland and Patterson 2002; Rumelhart and McClelland 1986). Unit-based models, typically attaining to connectionism, deny the existence of a system separating stems and affixes in the processing of inflected forms, and stipulate that all inflected forms (regular and irregular) are processed with the same system. Stressing the fact that tense acquisition in children is gradual, McClelland and Patterson (2002) defend the idea that the generation of inflected forms does not depend on an abstract rule, but rather on the child’s ability to combine phonological and semantic information. This type of approach explicitly states that the distinction between regular and irregular forms is not necessary, since a unified system can account for all verbs. The authors propose a connectionist model that generates inflected verbs based on regularities in the input. Interestingly, the authors stress that (p. 471) “contrary to some statements, connectionist networks are not simply analogy mechanisms that base their tendency to generalize on raw item-to-item similarity. Instead, they are sensitive to regularities, so that if an input–output relationship is fully regular, the network can closely approximate a categorical, symbolic rule”.

These types of connectionist models have been criticised for a number of reasons. As noted already by Massaro (1988), “connectionist models normally make unrealistic assumptions about the psychophysical relationships that are functional in the task” (p. 213). Marcus (2003) noted that most connectionist models cannot account for crucial properties of human cognition (and of human syntax), such as, for example, recursion. Bowers et al. (2009) explains that connectionist models are often too powerful and sensitive to statistical properties, in a way that does not mirror human cognition. However, despite this criticism, and contrary to general belief, sensitivity to rules is sometimes embedded in connectionist models. Roelofs (1997), for example, implemented a connectionist rule-based system of word-form encoding that is based on data obtained from cognitive studies on human participants. Connectionist models are not rule-based models because the input is not as regular as a rule would require. If it was, these models would be rule-based models.

One aspect that connectionist accounts seem to capture better than rule-based accounts is the relative pervasiveness of quasi-regular forms in language. As Seidenberg notes (2005), a substantial number of inflected forms do not fall within the regular pattern, nor do they behave as totally idiosyncratic forms: many forms are instead quasi-regular. This patterning is quite evident, for example, in the English past tense. Quasi-regular forms are forms obtained following a productive pattern, that is however less frequent than that normally labelled as regular (this type of label is however avoided in unit-based explanations, since the distinction between regular and irregular forms is refuted). Examples of these are: build/built, keep/kept, feel/felt and so on. Looking at the debate in hindsight, Seidenberg and Plaut (2014) notice that while a single analogical mechanism can account for the production and perception of these forms, rule-based theories (that propose that regulars and irregulars require two separate mechanisms, not a single one) require complex addenda to capture the phenomenon.

In addition, there are inclusive accounts that propose a redundant system in which rule-based explanations and unit-like explanations co-exist (Schreuder et al. 1999; Hay and Baayen 2005). These types of explanations successfully account for a large amount of evidence suggesting rule-based inflection and support the explanatory power of connectionist models, and account, for example, for the frequency effects observed in regular verbs. As Schreuder et al. (1999) explain, there is a fallacy in assuming that one system is incompatible with the other one. Our linguistic system may be redundant, operating thus with rules in combination with unit-like processes. A similar conclusion is proposed by Taft (2004). According to the author, unit-like and rule-like processes are active in parallel. In regular forms both processes successfully operate, while in irregular forms inflection is only attempted.

Whether or not rules are involved at all, both polarised and less polarised approaches agree on the existence of some form of sensitivity that speakers have toward inflectional morphology when it comes to the perception of inflected form. In fact, unit-like explanations (Seidenberg and Plaut 2014), rule-based explanations (Pinker and Ullman 2002) as well as redundant explanations (Hay and Baayen 2005) agree on the existence of a system that detects inflectional information. Whether this information is an emergent trait across a sample of occurrences (Seidenberg and Plaut 2014), a fully specified bound morpheme (Pinker and Ullman 2002) or the point of contrast occurring across different forms (Baayen et al. 2011, 2016), in all cases the speaker shows some kind of sensitivity that is then reflected in the following stages of speech mapping. In the present work we focussed our attention on this sensitivity, shifting the light from *real verbs with meaning* to *inflected forms with no meaning*. As such, our task is not bound to any specific theory, but it rather focuses on a phenomenon that is potentially predicted by different theories, but that was investigated so far only a few times, and to our knowledge only once in English.

### Sensitivity to Inflectional Morphology in Nonwords

Nonwords are a useful tool in psycholinguistic research because they allow for the study of phonological and morphological problems without activating semantic representations. Morphological (de)composition of regular verbs has long been investigated using nonwords. Seminal work by Berko (1958) shows that in production children and adults are able to apply inflectional morphemes to nonwords quite proficiently and from an early age. In her pioneering work, Berko (1958) elicited the plural of nonwords and showed that children as young as 4 years can apply the morphophonological rules needed to create the plural form. Similar results were obtained with verb inflection. In this version of the task children were presented with a subject (for example, a man exercising) and they were then told a few sentences such as the following (Berko 1958, p. 156):

This is a man who knows how to gling /gliŋ/. He is glinging. He did the same thing yesterday- What did he do yesterday? Yesterday he _______.

Evidence shows that children and adults are able to create the inflected forms of nonwords, and also that they are able to correctly chose the right allomorphic form (in recent times, Blything et al. (2018) have refined this classic finding, and showed that invented verbs are not always inflected using a regular form by children, and the probability of inflecting an invented verb as a regular depends on how similar this verb is to other existing verbs.)

Similar results were obtained in perception: in a visual word recognition task performed on Italian speaking participants, Caramazza et al. (1988) showed that morphological inflection of nonwords can apply also in perception. In their paper, the authors report a series of lexical decision tasks in which the participants were asked to decide whether a given stimulus was a word or a nonword. The stimuli created varied along two different variables: (1) they could start with an existing stem or a non-existing stem and (2) They could end with existing inflectional morphemes or with endings that are not inflectional morphemes in Italian. The authors reported that nonwords with possible inflectional morphemes took significantly longer to be discriminated than nonwords that could not be decomposed. In fact, nonwords that could not be decomposed into stems and inflectional morphemes were the quickest to be discriminated. Nonwords with partial morphological structure (either a real stem or a real affix) took slightly longer, and morphologically legal nonwords took the longest to be recognised.

It should be noted that participants were never presented with bare stems of any kind in this experiment. This made it unlikely for participants to be aware of the type of manipulation performed. Another novel aspect emerging from the work of Caramazza et al. (1988) is the idea that some form of inflection processing takes place even when we do not access the lexicon. This result is clearer than that observed in Berko (1958): while it may be argued that in Berko’s production task participants identified the nonwords with a specific item or action, this cannot be claimed in the tasks developed by Caramazza et al. (1988).

Clahsen et al. (1997) developed a series of tasks which support Caramazza et al. (1988)’s finding also for German. According to the authors, the tasks in German showed that nonwords, similarly to real words, get decomposed. Of particular interest for our work is the first experiment in Clahsen et al. (1997). In this task participants were familiarised with regularly inflected nonwords. Later, participants were asked to perform a lexical decision task using the same nonwords. These nonwords could either be regularly inflected (in a different tense than in the familiarisation phrase), or they could be irregularly inflected. Results showed that participants were significantly quicker in recognising the regularly inflected nonwords, suggesting that they were proficiently operating morphological inflection on the word forms. It may be noted that, contrary to Caramazza et al. (1988), and also contrary to the more recent study by Post et al. (2008) which is discussed below, the regularly inflected forms were slower to be recognized than the irregular ones. As the authors explain, this may be related to the fact that the irregular forms, due to the familiarisation phase, may have been perceived as illegal forms, while in Caramazza et al. (1988) and in Post et al. (2008), due to the absence of a familiarisation phase, the nonwords may have simply been perceived as irregulars.

In a more recent study, Post et al. (2008) reported similar results for English. Their experiment consisted of a minimal pairs discrimination task in which participants were asked to judge whether two words or nonwords presented in a pair were the same or different. The pairs consisted either of the stem and the inflected form, or of the first item repeated twice. The stimuli used were quite varied. The task contained several conditions, including real words with regular past (filled/fill), pseudo past regular words (mild/mile), plurals (meals/meal), nonwords ending in a potential past (gubbed/gub), nonwords not ending in the past (steet/stee). The results showed that participants took longer to differentiate potentially inflected nonwords (gubbed/gub) and inflected real words (filled/fill) from their stems than to differentiate nonwords without potential inflections (steet/stee).

A novel aspect in Post et al.’s work (2008) was that the task they developed better clarifies the relation between phonology and morphology in cueing the presence of potential inflections. Specifically, the work of Post et al. shows how the stem provides phonological cues to morphological inflection: it is not the nature of the affix per se that determines the RT (in fact, /t/ can be a bound morpheme in a different phonological context, namely following a devoiced word ending phoneme), but it is the relation between the final phoneme and the preceding segments that determines the RT. However, the stimuli used by Post et al. (2008) present a potential confound in that the minimal pairs compare inflected forms to bare stems. This is a strong cue to the nature of the manipulation performed in the test, and may bias the participants toward performing morphological decomposition. The task presented in this paper does not have this limitation. Instead of comparing inflected forms and their stems, our task always compares only inflected forms. In contrast to Post et al. (2008) the nonwords in our minimal pairs were always inflected. Participants were never presented with a noninflected stem, making it difficult or impossible for them to understand the type of manipulation provided. A further advantage of our task is the presence of a third set of nonwords, in which the two final phonemes are voiced, but the ending cannot be a bound morpheme (vɛlb/vɛlm). The presence of this condition is particularly important because it allows for the exclusion of a pure voicing explanation for longer reaction times in the condition with inflectional morphemes. If the morphological condition is not only slower than the non-morphological condition, but also than the control condition (vɛlb/vɛlm), voicing cannot be used as an explanation.

### Creation of the Materials

In this experiment, we investigated sensitivity to regular inflectional morphemes using nonwords. In English, regular verbs ending in /l/ take the /d/ ending when inflected in the past (e.g. kill–killed) and the ending /z/ when inflected in the third person present (e.g. kill–kills). Although morphological in other contexts, /t/ and /s/ do not bring grammatical information when following /l/. We compared discrimination of nonwords ending in /ld/ vs /lz/ and nonwords ending in /lt/ vs /ls/. All nonwords were deemed phonotactically legal using the Vitevitch and Luce (2004) calculator. Stimuli were created using the following procedure: first of all, 4 starting consonants were chosen. These were: /v/, /n/, /θ/, and /dʒ/. The choice was motivated by two factors:

1. All these consonants are allowed in word initial position in English, as is shown by the fact that the positional segment frequency value for these consonants in initial position is never zero. Positional segment frequency is a statistical measure obtained through the analysis of corpora. The measure indicates how often a specific phoneme appears in a specific position in words. For instance, /v/ has a positional segment frequency of 0.02 if used in word initial position. This means that /v/ appears in word initial position in 2 words out of 100. If /v/ was not allowed in word initial position in English, its positional segment frequency as a word beginning would have been zero. Positional segment frequencies for the word beginnings chosen for this study were never zero in the entire test. In addition, the consonants chosen have a relatively low frequency in word initial position. In fact, the values of positional segment frequencies vary between 0.02 and 0.006 for word initial phonemes in our test.
2. The choice of having word beginnings with a relatively low frequency was an advantage in terms of nonword generation. In fact, having infrequent word beginnings substantially reduced the risk of creating existing words.

All nonwords in this task are monosyllabic and as such there is only one vowel per word. The vowels used in this experiment are the following: /ɪ/, /aɪ/, /æ/, /ɔ/, /ʌ/. The choice of these vowels was motivated by biphone segment frequencies and positional segment frequencies. All these vowels are allowed in the second position of a word and all of them are allowed as the second phoneme of a biphone having any of the consonants presented above as a first phoneme. The fact that they are allowed in second position is demonstrated by the fact that the positional segment frequency of these vowels is never zero. The values can be checked in Cilibrasi (2016) (the positional segment frequency of the vowel is the second value from the left reported in each box). The fact that these vowels are allowed as members of a biphone having one of the consonants presented above in initial position is demonstrated by the fact that the biphone segment frequency of these biphones is never zero. The onset and nucleus of the nonwords were then combined with the potentially morphological codas presented at the beginning of this section: /lz/ and /ld/, and non-morphological codas, /ls/ and /lt/ (Table 1).

The nonwords were created using rules that allowed us to combine onsets, nuclei and codas. The productive rules for creating the nonwords was that each onset was combined with each nucleus. This enabled us to obtain 20 base forms. The four different codas were codas added to each base form, thus generating 80 nonwords. Forty of these contained potential inflectional morphemes, and 40 did not contain inflectional morphemes.

A third control condition was added to control for voicing effects. Without the third condition, the contrast between potentially morphosyntactic and non morphosyntactic minimal pairs could be explained by the fact that the two final phonemes in the first condition are both voiced, while the two final phonemes in the second condition are not. With the third condition we exclude this possibility. In the third condition, the two final phonemes are both voiced but they do not carry inflectional morphemes. The codas used in the control condition were the following: /lb/ and /lm/. The base forms were also applied to these codas to create the control condition, leading to a further 40 nonwords. Thus the final test contained 120 nonwords. A summary of the type of stimuli used in the test is presented in Table 1. The full list of stimuli is available in “Appendix” in phonetic transcription. In contrast to Post et al. (2008), all of our stems, in all conditions, ended in /l/, creating a very consistent pattern in which the 6 nonwords in the 3 conditions share the same stem, while in Post et al. (2008) stems were different across conditions. In short, our task includes the morphophonological sensitivity of Post et al. (2008), but it avoids the bare stem confound making it more similar to Caramazza et al. (1988).

**Table 1:** Materials

Condition	Morphological condition	Non-morphological condition	Phonological control condition
Examples	/vɔld/ /vɔlz/	/vɔlt/ /vɔls/	/vɔlb/ /vɔlm/
Manner of articulation	Plosive/fricative	Plosive/fricative	Plosive/nasal
Voicing	Voicing coherent	Voicing incoherent	Voicing coherent
Presence of inflectional morpheme	Present	Absent	Absent

Recording of the stimuli: the stimuli were recorded in the sound booth of the School of Psychology and Clinical Language Sciences of the University of Reading by a trained female linguist whose first language is English. The speaker received phonetic training and recorded with a Standard British pronunciation. The linguist was instructed to record stimuli in pairs. This corresponds to reading the words row by row in the phonetic list in “Appendix”.

The linguist was informed about the nature of the task, and she recorded pair by pair to ensure the recording of a subtle vowel lengthening in the morphological condition, as is typical in British speakers of English when producing inflected verbs [mean duration in ms (SD) for each condition: 224 (45), 146 (37), 178 (33)]. At a first sight this choice may appear unreasonable, as it looks like a choice that may raise the risk of a confound. However, this instruction is necessary if the aim is to use stimuli that would sound as similar as possible to “natural” inflected verbs. A relatively dark /l/ was used throughout the task, as is usual in Standard British English.

The software used was Audacity, running on a computer using Windows. The microphone was an AKG D80, the mixer was a Behringer Mini Mon, the pre-amplifier was a B-tech phono-microphone.

## Methods

Ethics, recruitment and consent: the current study was approved by the University of Reading Research Ethics Committee and it was given favourable opinion to proceed. The study was advertised on the University SONA system. Students received one credit for their participation in this study. Participants were allocated a numeric identifier which was used to anonymise the data. The information linking participants to this numeric identifier was stored in a separate and secure location.

Participants: twenty-two adult native speakers of British English were recruited. They were undergraduate students in Psychology, mean age 19 years and 10 months, Standard Deviation 1 year and 6 months. Nineteen were female, three were male;

Procedure: after signing the consent form, participants were given spoken instructions about the task by the researcher. The information they received was the following: “You are now going to hear pairs of made-up words. The two words presented in each pair may be identical or slightly different. Press white when you think the two words are identical, black when you think they are slightly different. Try to be as quick as you can”—After that, E-prime (Schneider et al. 2002) was launched and the participant was left alone in the room. Instructions on the screen guided the participant through the testing session. Participants were presented with a same/different task. The experiment was conducted using nonwords and contained 120 trials, 3 conditions and 60 items per condition. Each trial consisted of the presentation of two nonwords that could be either identical or that could differ in the final phoneme. The first slide was a fixation slide, lasting 1000 ms, containing only the symbol “+”, presented in white in the centre of a black screen. The second slide lasted 1000 s and corresponded to the presentation of the first nonword. While presenting the first nonword, the screen appeared completely black. The third slide contained the second nonword and it lasted 1000 ms. While presenting the second nonword the screen was completely black.

The pause between each pairs is about 500 ms (with some variation depending on the length of the second nonword). This pause is larger than in, for example, Post et al. (2008). This type of pause forces participants to recode speech at a more abstract phonological level (Pisoni 1973). During the presentation of this slide participants pressed “black” or “white” to express their judgment on the similarity of the nonwords. A white sticker was applied on the “w” key of the keyboard, and black sticker was applied on the “b” key of the keyboard. The fourth slide informed the participants that they were moving to the next trial, and was composed of an arrow presented in the centre of an otherwise black screen. The order of presentation of the trials was randomised for each participant.

Pairing of the items: in the task, half of the pairs contained the *same* nonword repeated twice, half contained two *different* nonwords. In the *same* pairs, the nonwords used were all of the nonwords ending in a plosive consonant. This corresponds in “Appendix” to all of the nonwords identified by an odd number. Since there are 60 odd numbers, this means that there were 60 trials in which participants were presented with the same nonword repeated twice. In the *different* pairs, participants were presented with a nonword ending in a plosive consonant, followed by its corresponding nasal or fricative. In “Appendix”, this corresponds to the pairing of any nonword identified with an odd number, and the nonword identified by the following even number. For example, nonword 13 was always followed by nonword 14, nonword 79 was always followed by nonword 80, and so on. Since there are 60 nonwords identified by odd numbers, and 60 corresponding nonwords identified with even numbers, this means that there were 60 trials in which participants were presented with two different nonwords. The second nonword in each pair was never a plosive. This choice was made because we wanted to make sure that the first word in the minimal pair was always priming some sort of *verb*-*like activation* in the morphological condition. If we used, for example, vɔlz as a prime for vɔlt we would have a further confound in the prime operated by vɔlz: apart from possibly being a verb inflected in the third person, vɔlz could also be a noun inflected in the plural. Using nonwords ending in /d/ as constant prime ensured the avoidance of this confound. To be consistent, the same pattern was kept for all of the three conditions, with the first nonword being always the one ending in a plosive consonant.

Scoring: E-prime was set to record the answer given (either “black”, “white”, a non-valid key or no answer). Reaction times were measured with the start of the second nonword. A no answer was coded when participants did not press any key for the entire duration of the third slide (1000 ms). For any type of given answer, E-prime measured the time (in ms) that the participants took to make their choice and press the button.

## Hypothesis and Predictions

Hypothesis: speakers are sensitive to the presence of inflectional morphology in the absence of meaning. More specifically:

1. The presence of inflectional morphology in nonwords makes judgments more challenging (Caramazza et al. 1988; Post et al. 2008).
2. It is easier to judge two identical items as “same” than to judge two slightly different items as “different” (McQueen and Cutler 1998; Beauvillain 1994).

### Prediction 1

Morphological minimal pairs would be more challenging than non-morphological minimal pairs

### Prediction 2

Same minimal pairs would be easier than different minimal pairs.

## Results

### Reaction Times

Data were analysed using linear mixed models (see Baayen 2010). Linear mixed models are a powerful statistical method that allows for the study of the variables that are manipulated and of random effects that are due to item idiosyncrasies or individual variation among participants. We constructed linear mixed models using the lmer function of the lme4 R package, version 1.1–7 (Bates et al. 2014). Reaction time was our response variable. The fixed factors were condition (morphological, non-morphological and control), type (whether the elements in the pair were same or different), and duration (the duration, in milliseconds, of the second nonword in each pair, see Bacovcin et al. 2017; Post et al. 2008, for a similar procedure). We centered the continuous variable “duration” to minimize collinearity between predictors. The model accounted for random effects by including participants, and item as random factors. Two participants were excluded due to a large number of missed answers (ids 18 and 22). Only correct answers were used in the analysis of reaction times (Table 2). We decided to delete from the analysis a selection of items that resemble irregular past tense verbs because these might have confounded our analysis. The items excluded from the analysis are: vɪlt, nɪlt, θɪlt and vɛlt.

**Table 2:** Mean reaction times

	Same pairs		Different pairs	
	Mean	SE	Mean	SE
Morphological	843	16	926	21
Non-morphological	778	16	802	14
Control	827	14	888	14

*SE* standard error

We followed a well-established procedure to obtain the most explanatory of the models (Pérez et al. 2016): keeping the full fixed structure, we looked for the best random structure using restricted maximum likelihood. Several different models were compared. All models had the same fixed structure (the interaction of condition, type and duration) and different combinations of the random structure that included the random variables (participant and item). In addition, we included models that tested random slopes. The full list of models compared is included in the “Appendix”. The model with the smallest AIC was chosen: cond * type * duration + (type|part) + (1|item).

As a following step we evaluated the optimal fixed structure by starting with the most complex one (the three-way interaction) to the simplest (a main effect) using stepwise model comparison (Pérez et al. 2016). The deletion of the three-way interaction led to a statistically significant difference, so we kept the full model. The final model contains thus condition, type and duration as fixed effects, and participants and item as random effects (Table 3). An ANOVA was then performed on the model. The analysis reveals that the three-way interaction, the two-way interactions and the main effects are all significant. Results are presented in the table below. The *p* values were provided by the ANOVA function of the lmerTest R package, version 2.0–11 (Kuznetsova et al. 2012), using the REML.

**Table 3:** Results of the ANOVA performed on the linear mixed model

	Mean square	NumDF	DenDF	F	*p*
cond	303,30	2	129.95	3.8891	< .001
type	101,626	1	147.62	13.03	< .001
duration	95,401	1	138.26	12.23	< .001
cond:type	23,446	2	129.85	3.006	0.05
cond:duration	17,524	2	128.85	2.24	0.1
type:duration	68,767	1	138.13	11.12	0.001
cond:type:duration	25,488	2	128.76	3.26	0.04

*cond* condition, *NumDF* numerator degrees of freedom, *DenDF* denominator degrees of freedom

The ANOVA shows a significant effect of condition, a significant effect of type, a significant effect of duration, a significant effect of the interaction between type and duration, and a significant effect of the interaction between condition, type and duration (Table 4).

**Table 4:** Summary of the linear mixed model effects for reaction times

	Estimate	SE	*df*	t value	*p*
Intercept	1168.65	196.90	243.55	5.93	< 0.001
cond2	− 447.75	218.79	193.21	− 2.04	0.04
cond3	− 465.45	214.86	229.29	− 2.16	0.03
typesame	− 577.47	217.19	202.15	− 2.65	0.008
duration	− 56.11	41.50	253.89	− 1.35	0.1
cond2:typesame	183.59	259.70	151.08	0.707	0.48
cond3:typesame	518.98	246.27	178.96	2.107	0.03
cond2:duration	74.96	47.27	191.96	1.58	0.11
cond3:duration	98.69	46.14	237.33	2.13	0.03
typesame:durat	119.42	46.86	202.86	2.54	0.01
cond2:typesame:duration	− 24.73	59.07	142.59	− 0.41	0.6
cond3:typesame:duration	− 116.01	54.51	175.83	− 2.128	0.03

*SE* standard error, *cond1* morphological condition, *cond2* non-morphological condition, *cond3* control condition, *df* degrees of freedom

**Fig. 1:**
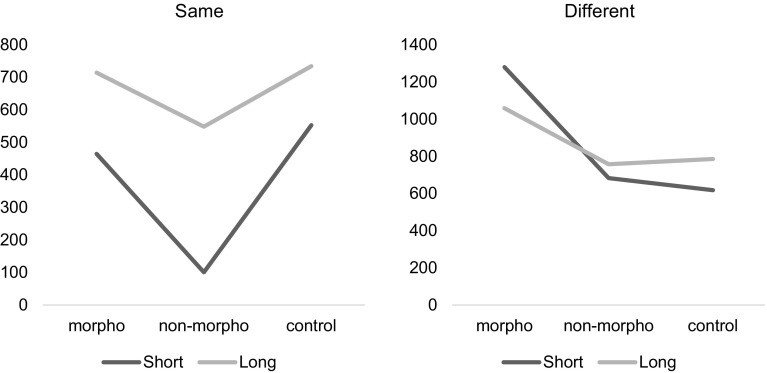
Reaction times. These graphs plot the predictions from coefficients in same and different minimal pairs

In order to understand the 3-way interaction we plotted the predictions from coefficients in Same and Different minimal pairs (Fig. 1). The graphs show that in the same subset performance does come down to duration (and voicing), with morphological and control conditions being slower than the non-morphological condition. In the different subset, instead, the morphological condition is considerably slower, and duration seems to play a minor role.

### Accuracy

Accuracy was analysed using a logistic regression. A forced entry method was used, as suggested by Field (2009) when assessing data that are testing a specific theory (compared to a stepwise analysis as it is suggested for data analysis that is not theory driven). In this type of analysis, variables involved in the model are chosen a priori, depending on the theoretical questions. We performed a logistic regression that included random factors using linear mixed models. For convergence reasons we kept the random structure in its simplest form. Following an established procedure (Pérez et al. 2016), we identified the best model by means of comparing the most complex model (three-way interaction) to gradually simpler models. Removal of the three-way interaction did not lead to a significant difference (*p* = 0.7) and the three-way interaction was then removed. Following the same procedure, we removed the interaction between type and duration (*p* = 0.8) and condition and duration (*p *= 0.1). The final model contained the interaction between condition and type, as well as the main effects of condition, type and duration: cond:type + cond + type + duration + (1|part) + (1|item).

The result of an ANOVA performed on the logistic regression using the final model is reported in the table below (Table 5).

**Table 5:** Results of the ANOVA performed on the logistic regression

	*df*	F	*p*
Condition	2	54.12	< 0.001
Type	1	46.96	< 0.001
Duration	1	41.87	< 0.001
Condition:type	2	6.69	< 0.05

*df* degrees of freedom

We found a significant effect of condition, a significant effect of duration, a significant effect of type and a significant interaction between condition and type.

**Fig. 2:**
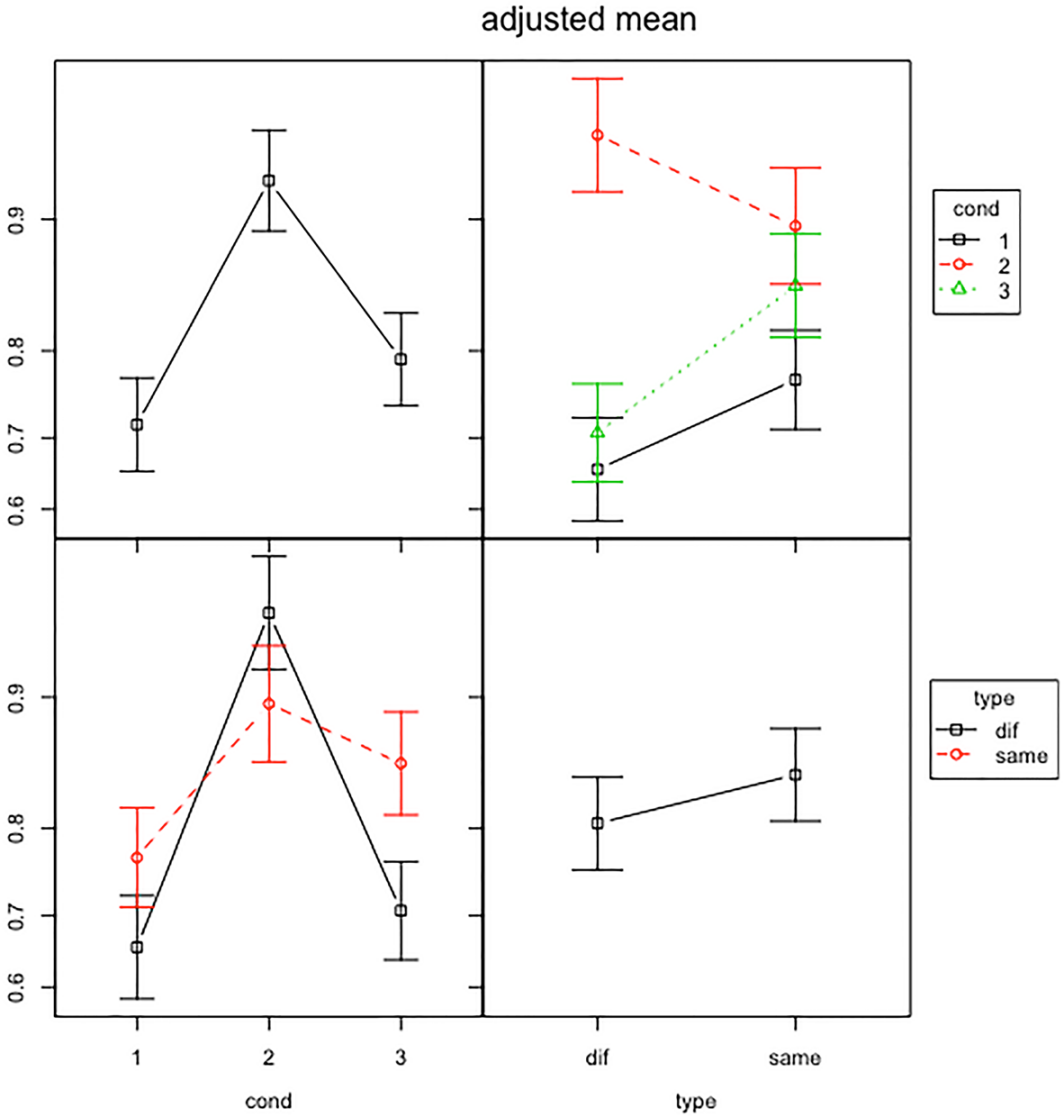
Accuracy. This graph plots the raw data (proportion of correct answers out of given answers) across different types (same/different) and across different conditions (morphological, non-morphological and control) as well as their interaction

An analysis of the fixed effects shows that condition 2 (non-morphological) differed from condition 1 (morphological), but condition 3 (control) did not differ from condition 1 (morphological). Same minimal pairs were more accurate than different minimal pairs in 2 conditions: the morphological and the control condition. In the non-morph condition, instead, the different minimal pairs were more accurate. We also observed a significant effect of duration, with long nonwords leading to more errors (Table 6). Since subjects were time-pressured in the completion of the task, this finding is expected (Fig. 2).

**Table 6:** Summary of the linear mixed model effects for accuracy

	Coefficient	SE	z	*p*
Intercept	6.23	0.984	6.336	< 0.001
cond2	2.06	0.292	7.056	< 0.001
cond3	0.22	0.234	0.965	0.33
typesame	0.55	0.243	2.276	0.02
duration	− 0.007	0.001	− 6.737	< 0.001
cond2:typesame	− 1.11	0.393	− 2.837	0.004
cond3:typesame	0.35	0.328	1.080	0.28

*SE* standard error, *cond1* morphological condition, *cond2* non-morphological condition, *cond3* control condition

## Discussion

This paper presents a novel task that aims to investigate whether English speakers are sensitive to the presence of inflectional morphology in nonwords. The task embeds the strengths of previously created tasks investigating the same issue, that is: (1) it has the morphophonological sensitivity of Post et al. (2008): the affix-like units used in our task are, as theirs, phonemes that could have been inflectional morphemes in a different phonological context; and (2) there are no bare stems, as in Caramazza et al. (1988), minimizing the possibility of participants to be aware of the type of manipulation performed.

**Fig. 3:**
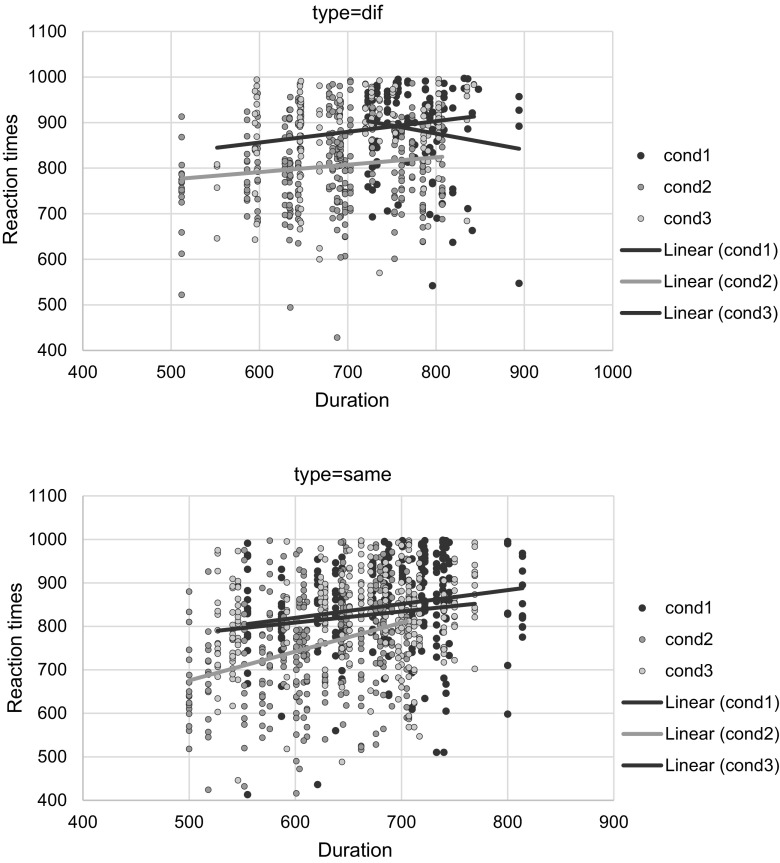
Scatterplots of item raw reaction-times/duration across condition in different and same minimal pairs

The first prediction, namely that morphological minimal pairs would be more challenging than non-morphological minimal pairs, was confirmed only in different minimal pairs (Fig. 3): when analysing reaction times for the discrimination of nonwords in the *different* subset, our results show that English speakers take longer to discriminate nonwords that contain potential inflectional morphemes, compared to both nonwords that do not contain them and nonwords in the phonological control condition. Our results are in line with previous data (Post et al. 2008; Caramazza et al. 1988). The data obtained in this study bring further evidence to the hypothesis that the presence of inflectional morphemes may be perceived even in the absence of meaning. In line with Post et al. (2008), our data show that the morphophonological context operates as a cue for the identification of inflectional morphemes. Nonwords with the specific morphophonological conditions defined by the rules of English regular tense inflection require a longer time to be recognised.

The first prediction is not confirmed in same minimal pairs. In this condition, reaction times revealed to be predicted by duration. A possible explanation for this result is that in same minimal pairs there is no need for morpheme detection, and thus speakers simply complete the task by comparing items as strings of sounds, independently from their internal morphological structure. In the different minimal pairs, instead, participants are pushed to find the element differentiating the two items, and when this difference consists of a possible bound morpheme, participants take longer. This interpretation of our results is line with Beauvillain (1994), who also failed to find morphological effects in the *same* subset of a same/different minimal pairs task with real words testing French speakers.

The analysis of the accuracy data shows that participants made more errors in the morphological condition than in the non-morphological condition. The contrasts between the morphological condition and the control condition, however, did not reach significance. This lack of significance may be attributed to a lack of power (considering that the direction is as expected), or it may be a more concerning effect of voicing (voiced minimal pairs are more difficult than unvoiced). Further studies should address this problem. In fact, this is the first study to add a condition that is matched on voicing features to the morphological condition but that does not contain morphological information, so also in relation to previous studies it is important to better characterise the role of voicing in the morphological effects so far reported.

Our results are not consistent with the data obtained by Clahsen et al. (1997) for German, but they are consistent with the interpretation proposed in their paper. In their study, Clahsen et al. (1997) showed that participants took a shorter amount of time to recognise nonwords which were inflected according to the regular rules of German, compared to the time they took to recognise nonwords that were modified with an inappropriate rule. The task performed by Clahsen et al. (1997) was however qualitatively very different, since participants were familiarised with the nonwords, and they were trained to recognise them as regular verbs. For this reason, the larger amount of time obtained with the non-regular inflections may be interpreted as a consequence of the fact that those nonwords were processed as a violation, rather than an irregular form.

The second prediction, namely that same minimal pairs would be easier than different minimal pairs, will be discussed only in relation to accuracy. This is because for reaction times *type* is part of the 3-way interaction, and so it is not appropriate to make a claim about its effect in isolation from condition and duration. When it comes to accuracy, the prediction was confirmed in two conditions: the morphological and the control condition. This finding is compatible with previous claims obtained from tasks on real words: the discrimination of related words is a particularly costly procedure, and it is thus a more challenging procedure than the recognition that one single item was repeated twice (McQueen and Cutler 1998; Beauvillain 1994; Jarvella and Meijers 1983). Interestingly, this contrast did not reach significance in Post et al. (2008), so this study may also be seen as better characterising the effect of type in comparison to previous work. It should be stressed that this finding was not reported in the non-morphological condition. This suggests that the voicing feature is interacting with type, and the facilitatory effect for *same* pairs may be modulated by phonological complexity (in this case applying only to nonwords with voiced endings). This finding may be a consequence of the different durations of these classes of consonants. When duration is shorter (as with the unvoiced consonants), the subject will resort less often to the purely acoustic comparison outlined in Beauvillain (1994), which leads to similar accuracy in same and different pairs of nonwords ending in unvoiced consonants. As Beauvillian explains (1994), the facilitation effect for same pairs is weaker when subjects attempt to encode the items, and we can reasonably assume that subjects will attempt to encode the item more often when the item is shorter. Nonetheless, further research is needed to assess the validity of this statement.

The data obtained with this test converge in the debate about morphological inflection. Our data suggest the presence of a morphophonological parser for inflectional morphemes. The parser uses phonological information and morphophonological properties as cues for the identification of inflectional morphemes. These data emphasise the importance of the relation between morphology and phonology. The idea that phonology and morphology intermingle in the identification of inflectional morphemes is certainly compatible with classic dual route models of tense inflection (such as Pinker and Ullman 2002 or Marslen-Wilson and Tyler 1997), although the notion was not necessarily stressed nor made explicit in these models. In fact, our data may be seen as shedding some light on the nature of the rule used in regular morphological inflection. This type of morphophonological explanation is particularly close to that of Post et al. (2008).

Our results may also be consistent with unit-like explanations, since our task shows that speakers are sensitive to inflectional morphemes, but it does not show anything about decomposition. Thus, in this sense, our results are compatible with proposals postulating analogy mechanisms, or spreading activation (Seidenberg and Plaut 2014), as long as sensitivity to inflectional morphemes is postulated as well.

Finally, our results are compatible with redundant models, such as Schreuder et al. (1999), according to which the processing of inflected forms normally requires two types of analysis operating at the same time: a rule-based parsing and a rote-based parsing. While both types of parsing operate in parallel, the authors assume a varying importance of each system, in relation to the frequency of the item presented. Frequent items tend to be parsed with the rote system, while infrequent items tend to be parsed with the rule system. With this type of redundant proposal, it becomes possible to accommodate the extensive amount of data that seem to confirm the existence of rule-like processes, as well as the important amount of data that shows frequency effects in regular verbs. This type of proposal makes a specific prediction about nonwords, since nonwords belong to the extreme of the low frequency distribution, with a frequency in the lexicon equalling zero. Our results are in line with the expectation of this model, since nonwords with morphological information took longer to process than the other nonwords in our task, in minimal pairs with different items.

Once again, it should be stressed that the parser revealed to be active only for the recognition of items in *different* minimal pairs, which suggests that specific conditions may be necessary for its activation. More specifically, these findings suggest that the parser operates in situations in which there is expectation or pressure for morphological detection, but it does not operate automatically and by default when presented with any string of sounds.

## Conclusion

This paper presented a novel task used to test English speakers’ sensitivity to the presence of inflectional morphemes in nonwords. The results show that participants are sensitive to the morphophonological cues that suggest the presence of inflectional morphemes when presented with different minimal pairs. Our results are in line with previous work on the topic. Specifically, our task confirms the findings of Caramazza et al. (1988), Clahsen (1999) and Post et al. (2008). The sum of the available literature suggests thus the presence of a system for morphological inflection available cross-linguistically that tunes to the morphophonological properties of each language.

This type of evidence is compatible with rule-like explanations of regular inflections (such as Pinker and Ullman 2002 or Marslen-Wilson and Tyler 1997), with unit-based explanations (such as the Seidenberg and Plaut 2014), and with redundant explanations (such as Schreuder et al. 1999).

## Appendix

Although the productive rules used ensured the generation of a large number of nonwords, a few nonwords were created with different vowels because the productive rules led to the generation of real words. Specifically, in the block starting with /dʒ/, /ɑ/ was chosen instead of /ɪ/ because the use of /ɪ/ would have led to /dʒɪlz/, which is an existing word (the plural of Jill, or the possessive form for Jill). In the block starting with /v/, /ɛ/ was used instead of /aɪ/ because using /ɛ/ would have led to one of the nonwords having one of its values of biphone segment frequency equalling zero (Cilibrasi 2016, p. 116).

See Tables 7 and 8.

**Table 7:** List of nonwords used in the task

Stem number	Morpho		Morpho		Non-morpho		Non-morpho		Control		Control	
1	1	vɪld	2	vɪlz	41	vɪlt	42	vɪls	81	vɪlb	82	vɪlm
2	3	vɛld	4	vɛlz	43	vɛlt	44	vɛls	83	vɛlb	84	vɛlm
3	5	væld	6	vælz	45	vælt	46	væls	85	vælb	86	vælm
4	7	vɔld	8	vɔlz	47	vɔlt	48	vɔls	87	vɔlb	88	vɔlm
5	9	vʌld	10	vʌlz	49	vʌlt	50	vʌls	89	vʌln	90	vʌlm
6	11	nɪld	12	nɪlz	51	nɪlt	52	nɪls	91	nɪlb	92	nɪlm
7	13	naɪld	14	naɪlz	53	naɪlt	54	naɪls	93	naɪlb	94	naɪlm
8	15	næld	16	nælz	55	nælt	56	næls	95	nælb	96	nælm
9	17	nɔld	18	nɔlz	57	nɔlt	58	nɔls	97	nɔlb	98	nɔlm
10	19	nʌld	20	nʌlz	59	nʌlt	60	nʌls	99	nʌlb	100	nʌlm
11	21	θɪld	22	θɪlz	61	θɪlt	62	θɪls	101	θɪlb	102	θɪlm
12	23	θaɪld	24	θaɪlz	63	θaɪlt	64	θaɪls	103	θaɪlb	104	θaɪlm
13	25	θæld	26	θælz	65	θælt	66	θæls	105	θælb	106	θælm
14	27	θɔld	28	θɔlz	67	θɔlt	68	θɔls	107	θɔlb	108	θɔlm
15	29	θʌld	30	θʌlz	69	θʌlt	70	θʌls	109	θʌlb	110	θʌlm
16	31	dʒald	32	dʒalz	71	dʒalt	72	dʒals	111	dʒalb	112	dʒalm
17	33	dʒaɪld	34	dʒaɪlz	73	dʒaɪlt	74	dʒaɪls	113	dʒaɪlb	114	dʒaɪlm
18	35	dʒæld	36	dʒælz	75	dʒælt	76	dʒæls	115	dʒælb	116	dʒælm
19	37	dʒɔld	38	dʒɔlz	77	dʒɔlt	78	dʒɔls	117	dʒɔlb	118	dʒɔlm
20	39	dʒʌld	40	dʒʌlz	79	dʒʌlt	80	dʒʌls	119	dʒʌlb	120	dʒʌlm

**Table 8:** List of models tested (only the first 4 converged and were compared)

	Models
M1	lmer(rt_corr ~ cond*type*length + (1|part) + (1|item), REML = TRUE, na.action = na.omit)
M2	lmer(rt_corr ~ cond*type*length + (cond|part) + (1|item), REML = TRUE, na.action = na.omit)
M3	lmer(rt_corr ~ cond*type*length + (type|part) + (1|item), REML = TRUE, na.action = na.omit)
M4	lmer(rt_corr ~ cond*type*length + (cond + type|part) + (1|item), REML = TRUE, na.action = na.omit)
M5	lmer(rt_corr ~ cond*type*length + (cond*type|part) + (1|item), REML = TRUE, na.action = na.omit)
M6	lmer(rt_corr ~ cond*type*length + (1|part) + (cond|item), REML = TRUE, na.action = na.omit)
M7	lmer(rt_corr ~ cond*type*length + (1|part) + (type|item), REML = TRUE, na.action = na.omit)
M8	lmer(rt_corr ~ cond*type*length + (1|part) + (cond + type|item), REML = TRUE, na.action = na.omit)
M9	lmer(rt_corr ~ cond*type*length + (1|part) + (cond*type|item), REML = TRUE, na.action = na.omit)
M10	lmer(rt_corr ~ cond*type*length + (cond|part) + (cond|item), REML = TRUE, na.action = na.omit)
M11	lmer(rt_corr ~ cond*type*length + (cond|part) + (type|item), REML = TRUE, na.action = na.omit)
M12	lmer(rt_corr ~ cond*type*length + (cond|part) + (cond + type|item), REML = TRUE, na.action = na.omit)
M13	lmer(rt_corr ~ cond*type*length + (cond|part) + (cond*type|item), REML = TRUE, na.action = na.omit)
M14	lmer(rt_corr ~ cond*type*length + (type|part) + (cond|item), REML = TRUE, na.action = na.omit)
M15	lmer(rt_corr ~ cond*type*length + (type|part) + (type|item), REML = TRUE, na.action = na.omit)
M16	lmer(rt_corr ~ cond*type*length + (type|part) + (cond + type|item), REML = TRUE, na.action = na.omit)
M17	lmer(rt_corr ~ cond*type*length + (type|part) + (cond*type|item), REML = TRUE, na.action = na.omit)
M18	lmer(rt_corr ~ cond*type*length + (cond + type|part) + (cond + type|item), REML = TRUE, na.action = na.omit)
M19	lmer(rt_corr ~ cond*type*length + (cond*type|part) + (cond*type|item), REML = TRUE, na.action = na.omit)
